# Characterization of odorant profiles and aroma characterization of vine tea (*Ampelopsis grossedentata*) using molecular sensory-omics

**DOI:** 10.1016/j.fochx.2025.103070

**Published:** 2025-09-22

**Authors:** Xiao Hua Chen, Yin Ku Liang, Fei Yan, Dong Qu, Wei Huang, Yifan Zhang, Tian Wan, Lin Jie Xi, Ching Yuan Hu

**Affiliations:** a College of Biological Science and Engineering, Shaanxi University of Technology, Hanzhong, Shaanxi 723001, China; b Shaanxi Provincial Key Laboratory of Bioresources, Shaanxi University of Technology, Hanzhong, Shaanxi 723001, China; c Qinling-Bashan Mountains Bioresources Comprehensive Development C. I. C., Hanzhong, Shaanxi 723001, China; d Qinba State Key Laboratory of Biological Resources and Ecological Environment, Shaanxi University of Technology, Hanzhong, Shaanxi 723001, China; e Hanzhong Food and Drug Inspection and Testing Center, Hanzhong, Shaanxi 723000, China; f Department of Human Nutrition, Food and Animal Sciences, College of Tropical Agriculture and Human Resources, University of Hawaii at Manoa, 1955 East-West Road, AgSci. 415J, Honolulu, HI 96822, USA

**Keywords:** Vine tea, Aroma profile, Quantitative descriptive analysis, Aroma reconstitution and omission experiments

## Abstract

The interest in vine tea and its extracts in the food industry has increased considerably due to its rich natural antioxidant dihydromyricetin and its non-toxic properties. Still, the strong herbal flavor makes it difficult for consumers to accept. This study aims to identify the odorant profile and aroma characterization of vine tea using molecular sensory-omics. The volatile compounds of vine tea were dominated by aldehydes, ketones and alcohols, and spathulenol and carotenoid-derived compounds were its critical characteristics. The aroma profile of vine tea was characterized by six aroma attributes, and twelve odorants were identified as the crucial contributors to them. Spathulenol played a vital role in the herbal aroma attribute. For aroma quality improvement, studies should focus on controlling the formation of compounds with herbal notes by aroma precursors, as well as eliminating the herbal note through process improvements. Our results provided experimental data for the implementation of these strategies.

## Highlights

• 99 volatile compounds in vine tea were detected by GC–MS, RI and standards.
• 12 odorants in vine tea were identified using GC-O and OAVs.
• Six aroma attributes for vine tea were identified by the QDA method.
• Spathulenol was identified as an important contributor to the herbal odor.

## 1 Introduction

Vine tea (*Ampelopsis grossedentata*) belongs to the family Vitaceae. It is also known as Teng Cha, Tuo Cha, or Duan Wu Cha in China, which is widely distributed in central and southern China, including Fujian, Jiangxi, Hubei, Shaanxi, Yunnan and Sichuan provinces (Fan et al., 2020). The vine's tender stems and leaves have long been used as herbal medicine to treat metabolic diseases, such as non-alcoholic fatty liver disease (NAFLD), thrombosis, diabetes, alcohol hangover, obesity, and heart disease (Carneiro et al., 2021; Hou et al., 2021; Sun et al., 2021; Tong et al., 2020). Recently, vine tea has received increasing attention in the food industry due to its rich natural antioxidant dihydromyricetin and its non-toxic properties. For example, vine tea and its extracts have been suggested to improve the shelf life and quality of foods such as edible oils, meat products, and breads (Ma et al., 2020). In addition, several studies have indicated that vine tea has potential applications in packaging and food safety due to its non-toxic properties and significant antibacterial and antioxidant activities (Zhang et al., 2021).

Vine tea is usually processed into a beverage for drinking using processing techniques similar to green tea, including withering, steaming, and drying processes (Carneiro et al., 2021). Aroma is a critical factor determining tea's quality and consumer acceptance (Yang et al., 2013). The acceptability of vine tea depends on its similarity to the flavor of the traditional teas, and the higher the similarity, the easier it is to be accepted (Lasekan & Lasekan, 2012). However, little is known about the aroma quality of vine tea extracts, which is crucial to determining whether it can be widely accepted as a food additive. A preliminary analysis of volatile compounds in vine tea was conducted by Carneiro et al. (2020); a total of 21 volatile compounds were detected, which were mainly composed of aldehydes and ketones. In addition, Li et al. (2024) identified 36 volatile compounds and 21 key aroma compounds from different parts of raw vine tea, which are mainly composed of aldehydes, ketones, esters and alcohols. Among them, the compounds with high concentration were dominated by geraniol, ethyl acetate, (*E*, *E*)-2,4-heptadienal, geranyl acetone, and (*E*)-2-hexenal. The odorants with high odor activity values (OAVs) were identified as ethyl acetate, (*E*, *Z*)-2,6-nonadienal, and geraniol. Volatile compounds are the foundation of aroma formation; most of them have also been reported in black, green, and oolong teas, indicating a certain similarity in aroma attributes between vine tea and traditional teas (Chen et al., 2019, Chen et al., 2020; Li et al., 2025). Although the results provide important data for us to understand the aroma of vine tea, the studies have not elucidated the differences in volatiles and sensory characteristics between vine tea and traditional teas, especially the relationship between sensory characteristics and chemical compounds, which is of great value for improving the aroma quality of vine tea. In this study, the volatiles of vine tea were extracted using SAFE. The volatile compounds profile was analyzed using *E*-nose and GC–MS, and the odorants were detected using GC-O and OAVs. The relationship between aroma characteristics and the volatile compound profile of vine tea was identified by aroma reconstitution and omission experiments. These results provide experimental data for a deeper understanding of the vine tea aroma and its further development and utilization.

## 2 Materials and methods

### 2.1 Chemicals and reagents

Internal standards of ethyl caprate (99.5 %) and n-Alkanes (C9-C27) were purchased from Sigma-Aldrich Corporation (Shanghai, China). Benzaldehyde (98 %), benzyl benzoate (99 %), benzyl salicylate (98 %), 2,4-dimethylbenzaldehyde (97 %), decanal (97 %), (*E*)-2-decenal (97 %), (*E*, *E*)-2,4-decadienal (95 %), dihydroactinidiolide (98 %), ethyl-benzaldehyde (97 %), furfural (99 %), hexanal (99 %), heptanal (98 %), (*E*)-2-heptenal (98 %), (*E*, *E*)-2,4-hexadienal (98 %), (*E*)-2-hexenal (99 %), (*E*, *E*)-2,4-heptadienal (98 %), (*Z*)-4-heptenal (99 %), isophytol (98 %), isovanillin (99 %), indole (99 %), methyl salicylate (99 %), methyl anthranilate (99 %), methyl hexadecanoate (97 %), 2-amylfuran (99 %), nonanal (98 %), (*E*)-2-nonenal (98 %), (*E*, *Z*)-2,6-nonadienal (98 %), (*E*)-2-octenal (98 %), (*E*, *E*)-2,4-octadienal (98 %), octanal (99 %), phytol (99 %), phenylacetaldehyde (98 %), paratolualdehyde (99 %), phenylethyl alcohol (98 %), safranal (99 %), 2-undecenal (98 %), undecanal (98 %), and 3Z-hexenyl benzoate (98 %) were purchased from J&K Scientific Ltd. (Beijing, China). 1-butanol (99 %), benzyl alcohol (99 %), β-caryophyllene (98 %), (*E*)-citral (98 %), citral (98 %), β-cyclocitral (97 %), 1,8-cineole (99 %), p-cymene (98 %), cinnamaldehyde (98 %), β-damascenone (99 %), dodecanoic acid (99 %), eugenol (98 %), farnesylacetone (98 %), geraniol (99 %), geranylacetone (98 %), geranyl linalool (98 %), hexahydrofarnesyl acetone (98 %), β-homocyclocitra (97 %),1-heptanol (99 %), 3-hepten-2-one (97 %), hexadecanoic acid (98 %), heptanoic acid (98 %), 1-hepten-3-one (98 %), hotrienol (98 %), (*Z*)-2-hexenol (98 %), (*Z*)-3-hexenol (98 %), hexahydropseudoionone (98 %), α-ionone (97 %), β-ionone (98 %), isothymol (98 %), limonene (99 %), trans-linalool oxide III and IV (mixture) (95 %), linalool (98 %), megastigmatrienone (98 %), 6-methyl-5-hepten-2-one (98 %), methyleugenol (98 %), nerol (98 %), trans-nerolidol (98 %), (*E*)-2-octenol (98 %), 1-octen-3-ol (98 %), 4-oxoisophorone (98 %), 3-octanone (98 %), sabinene (98 %), spathulenol (98 %), terpinolene (98 %), tetradecanoic acid (98 %), terpinen-4-ol (98 %), thymol (99 %), and α-terpineol (98 %) were from ANPEL Laboratory Technologies Inc. (Shanghai, China). Analytical grade methylene chloride and anhydrous sodium sulfate were from Sinopharm Chemical Reagents Co., Ltd. (Shanghai, China).

### 2.2 Sample preparation

**Fig. 1:**
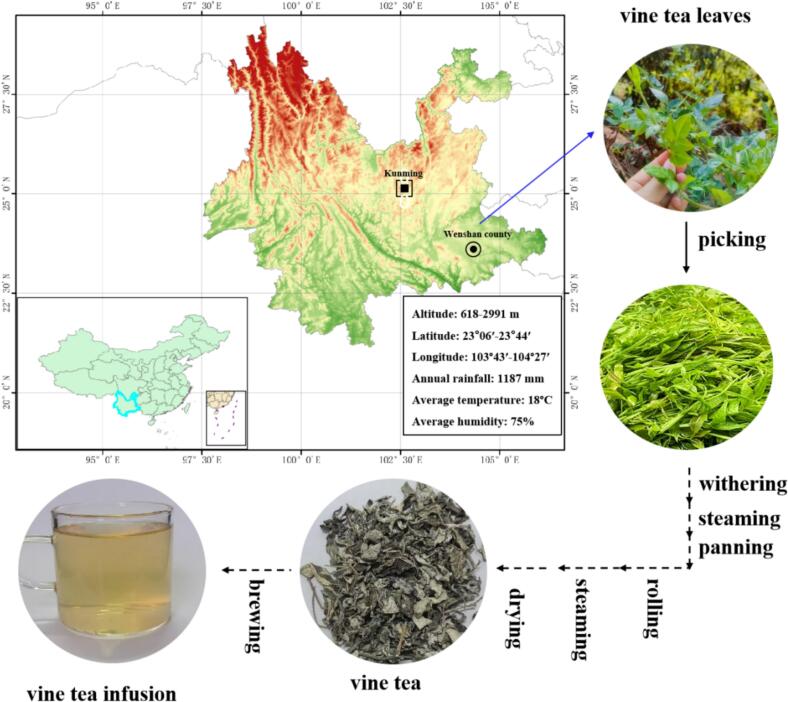
Vine tea sample information.

The fresh vine tea leaves were picked from Jiu Long Mountain in Wenshan County of Yunnan Province (Fig. 1). The leaves were exposed to sunlight at room temperature, about 25 °C, for 24 h. They were then steamed in boiling water for 2 min. After that, the leaves were panned at 150 °C for 20 min. Then, the tea leaves were rolled in a rolling machine at 25 °C for 60 min. The rolled leaves were steamed again in a steamer at 50 °C for 60 min. Finally, the leaves were dried at 70 °C for 12 h to obtain vine tea.

### 2.3 Extraction of volatiles

The solvent-assisted flavor evaporation (SAFE) method described by Schuh and Schieberle (2006) was used to prepare aroma extracts. Briefly, 70.0 g of vine tea was brewed in hot water (90 °C, 1.5 L) for 5 min, and then the tea infusion was collected and cooled to room temperature. After that, the tea infusion was extracted with 100 mL of methylene chloride four times. Finally, the organic phases were distilled by the SAFE technique at 35 °C and concentrated to 1.0 mL by vacuum distillation and a gentle nitrogen stream for the subsequent experiments.

### 2.4 GC–MS-RI analysis

A GC2010plus-TQ8040MS/MS (Shimadzu Technologies, Tokyo, Japan) with InertCap-purWax (30 m × 0.25 mm i.d. × 0.25 μm film thickness; Shimadzu Technologies, Tokyo, Japan) was used to analyze the aroma samples. The initial oven temperature was 40 °C, held for 5 min, ramped to 220 °C at a rate of 3 °C/min, and held for 5 min at this final temperature. The injection mode was splitless. The injector, transfer line, and ion source temperatures were 200 °C, 230 °C, and 230 °C, respectively. The mass range was 40 to 500 *m*/*z* in full scan mode. The retention indices (RI) were calculated using the method reported by Chen et al. (2019):

(1) $$\mathrm{RI}=100\times n+100\times \left[\mathrm{RT}\left(x\right)-\mathrm{RT}\left(n\right)\right]/\left[\mathrm{RT}\left(n+1\right)\;\mathrm{RT}\left(n\right)\right]$$

Where RT(x) is the retention time of compound x; RT(n) and RT(n + 1) are the retention times of the alkanes with carbon number n and n + 1 immediately eluting before and after the compound x, respectively.

Each volatile compound was identified by comparing its mass spectra, retention index (RI), and odor quality to authentic compounds. Each volatile compound was quantified using an internal standard method, with 2-octanol (5 μg/mL) as the internal standard compound. The response factor (Rf) was calculated following the method described by Zhang et al. (2015).

(2) $$\mathrm{Rf}=A_{\mathrm{IS}}\times m_{a}/m_{\mathrm{IS}}\times A_{a}$$

Where *A*_*IS*_ and *A*_*a*_ are the peak areas of internal standard compound (IS) and analyte (a), respectively; *m*_*IS*_ and *m*_*a*_ are the mass of internal standard compound (IS) and analyte (a), respectively.

### 2.5 *E*-nose analysis

A SuperNose electronic nose system (ISENSO Co., France) with a sampling apparatus, detector unit, and pattern recognition software was employed for volatiles profile analysis. The detector unit contains ten different metal oxide sensors including S1 (alkanes), S2 (alcohols, aldehydes, short-chain alkanes), S3 (ozone), S4 (sulfur-containing organics), S5 (nitrogen oxides), S6 (phenylketones, alcohols and aldehydes, aromatics), S7 (ketones and alcohols), S8 (short-chain alkanes), S9 (organic solvents), and S10 (hydrogen). The aroma analysis was programmed according to the procedure described by Peng et al. (2023). The dry vine tea (5 g) was brewed for 5 min in hot water (95 °C; 300 mL). Subsequently, the residue was separated by filtration, and the infusion was used for *E*-nose analysis. The sample preparation time and automatic zeroing time were both 5 s, the detection time was 60 s, and the interval for data collection was 1 s.

### 2.6 GC-O and OAVs analysis

The odorants were detected using a gas chromatography-flame ionization detector (GC2010plus-FID, Shimadzu, Tokyo, Japan) -olfactometry (OPV277, Shimadzu, Tokyo, Japan) system. The chromatographic conditions were the same as described in section 2.4. The FID and sniffing port temperatures were 250 °C and 170 °C, respectively. The split ratio between FID and olfactometry was 1:1. Eleven experienced panelists (staff and undergraduate students from the Shaanxi University of Technology Food Science and Engineering Department) were recruited for GC-O analysis. They were asked to record each perceived odor attribute and its intensity during the entire running time of GC-O analysis. The odor intensity was evaluated using a five-point scale from 1 (weak) to 5 (strong). The OAVs of aroma compounds were the concentration ratios to their odor threshold.

### 2.7 Aroma profile analysis

The aroma profile of vine tea infusion was evaluated using a quantitative descriptive analysis (QDA) method, which has gained acceptance for sensory evaluation of various foods (Niu et al., 2025). The vine tea infusion was prepared using the method described in section 2.4 and provided to the eleven panelists mentioned in section 2.6. They were asked to provide sensory attribute terms for vine tea infusion. Subsequently, the terms were discussed to determine their similarities and differences in the first group session. The redundant and ambiguous terms were eliminated and modified in the second discussion. The sensory terms for vine tea infusion were preliminarily determined following the third group session. In the fourth session, six main sensory attributes (green/grassy, floral, herbal, fruity, woody and honey-like) were defined, and the reference standards were also brought in, which were used to provide clear examples of individual terms (Table 1). After that, the intensity of the defined terms was quantified using a linear five-point scale from 1 (weak) to 5 (strong) in vine tea infusion. Each term's final sensory score was the average of the scores from the panelists and was used to plot in a radar diagram.

**Table 1:** The terms for sensory attributes of vine tea were developed and defined through quantitative descriptive analysis.

Sensory terms	Definition	Sensory scores	Reference standards
Green/grassy	Tender grass and hay odor notes	4.0 ± 0.6	90 mg/L leaf alcohol aqueous solution
Floral	Aroma related to the floral note	2.0 ± 0.6	100 mg/L linalool aqueous solution
Herbal	Aroma related to the herb	3.8 ± 0.8	3.0 g of ginseng chips in 200 mL of aqueous solution
Fruity	Aroma related to ripe fruit	1.5 ± 0.5	0.1 mg/L β-damascenone aqueous solution
Woody	Aroma related to wood	2.4 ± 0.5	0.1 mg/L β-ionone in aqueous solution
Honey-like	Aroma related to honey	1.0 ± 0.0	0.5 mg/L phenylacetaldehyde aqueous solution

The experimental procedures used in this study were approved by the Management Committee of Shaanxi Provincial Key Laboratory of Bioresources (Approval No. 2024–07). All sensory panelists have read and signed the consent form before participating in this study. All experiments were conducted strictly in accordance with the regulations of the Shaanxi University of Technology and the guidelines proposed in the Helsinki Declaration.

### 2.8 Reconstitution and omission experiments

The reconstitution experiment was performed according to the method proposed by Schuh and Schieberle (2006). The vine tea infusion was prepared using the method described in section 2.4. Synchronously, the vine tea aroma model was set up by dissolving the twelve compounds in water using the same concentrations as listed in Table 2: (*Z*)-4-heptenal, octanal, phenylacetaldehyde, (*E*)-2-octenal, (*E*, *Z*)-2,6-nonadienal, (*E*)-2-nonenal, decanal, (*E*, *E*)-2,4-decadienal, β-damascenone, α-ionone, β-ionone, and spathulenol.

**Table 2:** Chemical names and concentrations of volatile compounds in vine tea.

No.	RI	Compounds	Concentration (μg/kg)	Rf	Identification	Origin
1	810	Hexanal	5.4 ± 0.4	1.124	RI, Std, MS	Lipid oxidation
2	851	(*E*)-2-hexenal	46.7 ± 8.1	0.985	RI, Std, MS	Lipid oxidation
3	892	(*Z*)-4-heptenal	2.1 ± 0.0	1.459	RI, Std, MS	Lipid oxidation
4	894	Heptanal	1.3 ± 0.5	1.500	RI, Std, MS	Lipid oxidation
5	901	(*E*, *E*)-2,4-hexadienal	0.7 ± 0.3	0.899	RI, Std, MS	Lipid oxidation
6	949	(*E*)-2-heptenal	1.4 ± 0.2	1.235	RI, Std, MS	Lipid oxidation
7	951	Benzaldehyde	2.4 ± 0.0	1.079	RI, Std, MS	Amino acid degradation
8	999	Octanal	0.6 ± 0.0	1.400	RI, Std, MS	Lipid oxidation
9	1006	(*E*, *E*)-2,4-heptadienal	21.8 ± 2.1	1.001	RI, Std, MS	Lipid oxidation
10	1038	Phenylacetaldehyde	7.4 ± 1.6	1.612	RI, Std, MS	Amino acid degradation
11	1053	(*E*)-2-octenal	3.1 ± 0.0	0.941	RI, Std, MS	Lipid oxidation
12	1061	Paratolualdehyde	0.5 ± 0.1	1.392	RI, Std, MS	Amino acid degradation
13	1100	Nonanal	3.4 ± 0.5	0.980	RI, Std, MS	Lipid oxidation
14	1104	(*E*, *E*)-2,4-octadienal	1.0 ± 0.7	0.571	RI, Std, MS	Lipid oxidation
15	1148	(*E*, *Z*)-2,6-nonadienal	1.6 ± 0.2	1.245	RI, Std, MS	Lipid oxidation
16	1155	(*E*)-2-nonenal	0.6 ± 0.4	1.238	RI, Std, MS	Lipid oxidation
17	1157	Ethyl-benzaldehyde	0.2 ± 0.0	0.850	RI, Std, MS	Amino acid degradation
18	1169	2,4-Dimethylbenzaldehyde	1.8 ± 0.7	0.892	RI, Std, MS	Amino acid degradation
19	1194	Safranal	1.9 ± 0.9	1.469	RI, Std, MS	Carotenoids degradation
20	1201	Decanal	0.9 ± 0.3	0.982	RI, Std, MS	Lipid oxidation
21	1257	(*E*)-2-decenal	1.4 ± 0.5	0.951	RI, Std, MS	Lipid oxidation
22	1288	(*E*, *E*)-2,4-decadienal	0.8 ± 0.3	1.665	RI, Std, MS	Lipid oxidation
23	1359	2-Undecenal	0.3 ± 0.0	1.360	RI, Std, MS	Lipid oxidation
24	1392	Isovanillin	0.1 ± 0.0	0.750	RI, Std, MS	Amino acid degradation
25	1404	Undecanal	0.4 ± 0.1	0.924	RI, Std, MS	Lipid oxidation
26	1606	Cinnamaldehyde	0.4 ± 0.0	1.360	RI, Std, MS	Amino acid degradation
		**Aldehydes**	104.2 ± 6.1			
27	929	3-Hepten-2-one	0.1 ± 0.0	1.506	RI, Std, MS	Lipid oxidation
28	973	1-Hepten-3-one	0.3 ± 0.0	1.380	RI, Std, MS	Lipid oxidation
29	982	6-Methyl-5-hepten-2-one	8.8 ± 2.8	0.962	RI, Std, MS	Carotenoids degradation
30	1138	4-Oxoisophorone	0.3 ± 0.1	0.948	RI, Std, MS	Carotenoids degradation
31	1239	3-Octanone	0.9 ± 0.2	1.633	RI, Std, MS	Lipid oxidation
32	1380	β-Damascenone	0.3 ± 0.1	1.825	RI, Std, MS	Carotenoids degradation
33	1400	Hexahydropseudoionone	0.7 ± 0.2	1.254	RI, Std, MS	Carotenoids degradation
34	1423	α-Ionone	16.3 ± 2.4	1.279	RI, Std, MS	Carotenoids degradation
35	1427	Dehydro-β-ionone	1.3 ± 0.3	1.163	MS	Carotenoids degradation
36	1448	Geranylacetone	28.7 ± 3.8	0.817	RI, Std, MS	Carotenoids degradation
37	1482	β-Ionone	25.8 ± 3.1	1.114	RI, Std, MS	Carotenoids degradation
38	1620	Megastigmatrienone	0.3 ± 0.1	0.966	RI, Std, MS	Carotenoids degradation
39	1839	Hexahydrofarnesyl acetone	16.7 ± 2.2	1.069	RI, Std, MS	Carotenoids degradation
40	1913	Farnesylacetone	102.2 ± 6.1	0.950	RI, Std, MS	Carotenoids degradation
		**Ketones**	118.7 ± 2.7			
41	854	(*Z*)-3-hexenol	2.0 ± 0.4	1.548	RI, Std, MS	Lipid oxidation
42	864	(*Z*)-2-hexenol	11.6 ± 1.8	1.077	RI, Std, MS	Lipid oxidation
43	866	1-Butanol	0.3 ± 0.0	1.633	RI, Std, MS	Lipid oxidation
44	965	1-Heptanol	0.1 ± 0.0	1.931	RI, Std, MS	Lipid oxidation
45	975	1-Octen-3-ol	2.2 ± 0.1	1.667	RI, Std, MS	Lipid oxidation
46	1030	Benzyl alcohol	0.3 ± 0.0	1.485	RI, Std, MS	Amino acid degradation
47	1064	(*E*)-2-octenol	1.1 ± 0.0	1.559	RI, Std, MS	Lipid oxidation
48	1108	Phenylethyl alcohol	0.3 ± 0.0	0.930	RI, Std, MS	Amino acid degradation
49	1942	Isophytol	0.6 ± 0.1	0.895	RI, Std, MS	Chlorophyll hydrolysis
50	2108	Phytol	32.4 ± 3.0	0.966	RI, Std, MS	Chlorophyll hydrolysis
		**Alcohols**	50.7 ± 2.5			
51	925	α-Thujene	0.3 ± 0.1	/	MS	Terpenoid pathway
52	969	β-Thujene	0.1 ± 0.0	/	MS	Terpenoid pathway
53	1013	Sabinene	0.2 ± 0.0	1.506	RI, Std, MS	Terpenoid pathway
54	1019	p-Cymene	0.2 ± 0.1	1.292	RI, Std, MS	Terpenoid pathway
55	1024	Limonene	1.9 ± 1.0	1.068	RI, Std, MS	Terpenoid pathway
56	1026	1,8-Cineole	2.0 ± 0.3	1.595	RI, Std, MS	Terpenoid pathway
57	1075	3-p-Menthene	0.6 ± 0.0	/	MS	Terpenoid pathway
58	1093	cis-p-Menth-2-en-1-ol	0.3 ± 0.2	/	MS	Terpenoid pathway
59	1097	Linalool	2.0 ± 0.0	1.494	RI, Std, MS	Terpenoid pathway
60	1084	*Trans*-linalool oxide III	0.2 ± 0.1	1.353	RI, Std, MS	Terpenoid pathway
61	1165	*Trans*-linalool oxide IV	0.2 ± 0.1	0.902	RI, Std, MS	Terpenoid pathway
62	1172	Terpinen-4-ol	0.6 ± 0.1	1.335	RI, Std, MS	Terpenoid pathway
63	1180	Thymol	0.5 ± 0.1	1.497	RI, Std, MS	Terpenoid pathway
64	1185	Hotrienol	0.7 ± 0.2	0.868	RI, Std, MS	Terpenoid pathway
65	1186	α-Terpineol	1.7 ± 0.0	4.173	RI, Std, MS	Terpenoid pathway
66	1215	β-Cyclocitral	7.3 ± 1.8	0.857	RI, Std, MS	Terpenoid pathway
67	1224	Nerol	0.4 ± 0.2	1.498	RI, Std, MS	Terpenoid pathway
68	1237	(E)-Citral	0.5 ± 0.3	1.600	RI, Std, MS	Terpenoid pathway
69	1251	Geraniol	1.5 ± 0.8	0.845	RI, Std, MS	Terpenoid pathway
70	1253	β-Homocyclocitra	0.9 ± 0.1	1.094	RI, Std, MS	Terpenoid pathway
71	1265	Terpinolene	0.5 ± 0.3	1.162	RI, Std, MS	Terpenoid pathway
72	1266	Citral	0.8 ± 0.0	1.013	RI, Std, MS	Terpenoid pathway
73	1308	Isothymol	0.2 ± 0.0	1.210	RI, Std, MS	Terpenoid pathway
74	1352	Eugenol	0.2 ± 0.1	1.053	RI, Std, MS	Terpenoid pathway
75	1416	β-Caryophyllene	0.4 ± 0.2	1.069	RI, Std, MS	Terpenoid pathway
76	1473	γ-Cadinene	0.1 ± 0.0	/	MS	Terpenoid pathway
77	1492	Methyleugenol	0.1 ± 0.0	1.485	RI, Std, MS	Terpenoid pathway
78	1504	Farnesene isomer	0.1 ± 0.0	1.577	MS	Terpenoid pathway
79	1540	α-Calacorene	0.1 ± 0.0	0.842	MS	Terpenoid pathway
80	1546	α-Eudesmol	0.4 ± 0.2	1.203	MS	Terpenoid pathway
81	1559	*Trans*-nerolidol	6.3 ± 1.5	1.190	RI, Std, MS	Carotenoids degradation
82	1575	Spathulenol	14.3 ± 2.6	1.157	RI, Std, MS	Terpenoid pathway
83	1580	Caryophyllenoxide	4.6 ± 0.4	/	MS	Terpenoid pathway
84	1639	α-Cadinol	0.3 ± 0.0	/	MS	Terpenoid pathway
85	2024	Geranyl linalool	0.4 ± 0.0	0.954	RI, Std, MS	Terpenoid pathway
		**Terpenes**	24.8 ± 1.4			
86	981	Heptanoic acid	0.8 ± 0.4	1.367	RI, Std, MS	Lipid oxidation
87	1558	Dodecanoic acid	2.6 ± 1.1	1.583	RI, Std, MS	Fatty acid
88	1756	Tetradecanoic acid	4.3 ± 1.4	0.948	RI, Std, MS	Fatty acid
89	1975	Hexadecanoic acid	55.0 ± 6.0	1.392	RI, Std, MS	Fatty acid
		**Acids**	62.7 ± 5.4			
90	1189	Methyl salicylate	0.8 ± 0.2	1.072	RI, Std, MS	esterification
91	1335	Methyl anthranilate	0.3 ± 0.0	1.292	RI, Std, MS	esterification
92	1524	Dihydroactinidiolide	3.0 ± 0.0	1.536	RI, Std, MS	esterification
93	1566	3*Z*-hexenyl benzoate	1.1 ± 0.3	0.939	RI, Std, MS	esterification
94	1759	Benzyl benzoate	0.3 ± 0.1	1.331	RI, Std, MS	esterification
95	1863	Benzyl salicylate	0.2 ± 0.0	1.168	RI, Std, MS	esterification
96	1920	Methyl hexadecanoate	3.4 ± 0.7	1.289	RI, Std, MS	esterification
		**Esters**	9.1 ± 0.2			
97	834	Furfural	0.5 ± 0.2	1.474	RI, Std, MS	Maillard reaction
98	987	2-Amylfuran	0.8 ± 0.3	1.192	RI, Std, MS	Maillard reaction
99	1287	Indole	0.1 ± 0.0	1.274	RI, Std, MS	Amino acid degradation
		**Heterocycles**	1.4 ± 0.1			

No., peak number; RI, retention index; Rf, response factor relative to internal standard; Std, confirmed by authentic standards; MS, mass spectrum comparison using Nist library; “/,” not detected.

The eleven panelists were given the vine tea infusion and the aroma reconstitution model. They were asked to rate the intensities of the given odor attributes on a linear five-point scale from 1 (weak) to 5 (strong).

An omission model was prepared by removing spathulenol based on the reconstitution model and was used to verify the role of spathulenol in vine tea infusion aroma. The panelists were asked to compare the differences in sensory attributes between reconstitution and omission models using the method mentioned earlier.

### 2.9 Statistical analysis

All tests were repeated three times, and the final results were expressed as mean ± standard deviation. The difference (*P* < 0.05) among the volatile compound profiles was evaluated using one-way analysis of variance (ANOVA) by Duncan's

multiple comparison tests in SPSS Statistics 19.0 software (SPSS Inc., Chicago, IL, USA). The correlation analysis was performed using Originpro 2021 (OriginLab, Northampton, MA).

## 3 Results and discussion

### 3.1 Volatile compounds profile of vine tea

#### 3.1.1 *E*-nose analysis

**Fig. 2:**
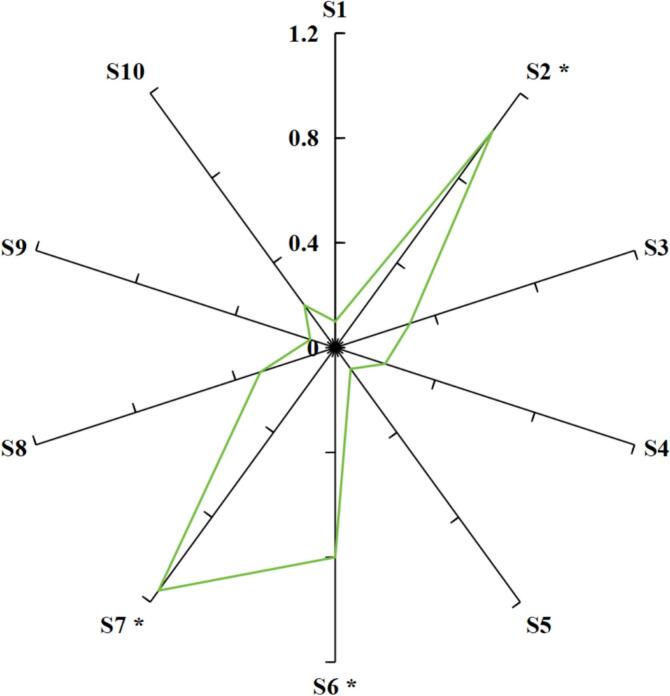
Volatile compounds profile of vine tea infusion based on *E*-nose data (*P*<0.05).

*E*-noses have been widely used in classifying the grade level of foods, discriminating the quality and sensory characteristics due to their rapid, convenient and accurate advantages (Shao et al., 2018). The stronger the response value of the sensor, the higher the abundance of volatile compounds sensitive to the sensor (Siripatrawan & Harte, 2015). As given in Fig. 2, S2 (sensitive to alcohols, aldehydes, short-chain alkanes), S6 (sensitive to phenylketones, alcohols and aldehydes, aromatics) and S7 (sensitive to ketones and alcohols) sensors had strong response to volatile compounds of samples, indicating that the vine tea might contain more abundances of aldehydes, ketones and alcohols.

Previous studies have also reported the E-nose's efficient differentiation of volatile compound profiles. For example, the high abundance of aromatic and alcohol compounds was detected simultaneously by E-nose and GC–MS analysis in mulberry tea infusion (Ruengdech & Siripatrawan, 2020). The GC–MS was performed to further understand the profile of volatile compounds.

#### 3.1.2 GC–MS analysis

As shown in Table 1, a total of 99 volatile compounds were detected in the vine tea, and most of them were composed of terpenes (35), aldehydes (26), ketones (14), alcohols (10), esters (7), acids (4) and heterocycles (3). Ketones were the most abundant volatile compounds, accounting for 29.3 % of the total compound content. The high content of ketones was dominated by farnesylacetone (102.2 ± 2.5 μg/kg), followed by geranylacetone (28.7 ± 5.7 μg/kg), β-ionone (25.8 ± 3.0 μg/kg), hexahydrofarnesyl acetone (16.7 ± 2.8 μg/kg), α-ionone (16.3 ± 3.6 μg/kg) and 6-methyl-5-hepten-2-one (8.8 ± 1.1 μg/kg). Ketones mainly originated from carotenoid degradation. For example, farnesylacetone and hexahydrofarnesyl acetone come from the oxidative cleavage of phytoene (Yahyaa et al., 2013). α-ionone and β-ionone were produced through the symmetric cleavage of carotenoids by carotenoid dioxygenase 1 (Ahrazem et al., 2016). β-damascenone comes from the enzymatic oxidation of neoxanthin (Huang et al., 2009). Photooxidation of phytofluene results in geranylacetone and 6-methyl-5-hepten-2-one (Ho et al., 2015). Hexahydropseudoionone and dehydro-β-ionone in Table 2 were related to the photooxidation of β-carotene induced by ultraviolet light (Baldermann et al., 2010). Carotenoids are commonly present in all photosynthetic organisms (bacteria, algae, and plants), as well as some non-photosynthetic bacteria and fungi. As an auxiliary photosynthetic pigment, its biosynthesis is stimulated and regulated by light (Nisar et al., 2015). Therefore, these volatile compounds' extensive formation and accumulation might be related to the strong ultraviolet environment in which vine tea grows.

Aldehydes were the second most abundant group of volatile compounds, accounting for 25.7 % of the total compound content. The (*E*)-2-hexenal (46.7 ± 8.4 μg/kg) was identified with significantly high contents, followed by (*E*, *E*)-2,4-heptadienal (21.8 ± 2.1 μg/kg). In addition, hexanal (5.4 ± 0.4 μg/kg), phenylacetaldehyde (7.4 ± 1.6 μg/kg), nonanal (3.4 ± 0.5 μg/kg) and (*Z*)-4-heptenal (2.1 ± 0.0 μg/kg) also presented at moderate levels. They were generated through lipid oxidation, carotenoid degradation and amino acid decomposition. For example, (*E*)-2-hexenal and hexanal originated from the lipoxygenase-mediated α-linolenic acid and linolenic acid oxidation, respectively (Yang et al., 2013). Lipid degradation can also produce (*E*, *E*)-2,4-heptadienal, nonanal, (*Z*)-4-heptenal, heptanal, (*E*)-2-heptenal, octanal, (*E*, *E*)-2,4-octadienal, (*E*, *Z*)-2,6-nonadienal and (*E*)-2-nonenal (Ho et al., 2015). While Safranal and phenylacetaldehyde are formed from the degradation of carotenoids and amino acids, respectively (Yang et al., 2013).

Acids accounted for 15.5 % of the total volatile compound content. Four acids were detected, most of which were fatty acids. Among them, hexadecanoic acid exhibited a very high level with a concentration of 55.0 ± 6.0 μg/kg. Alcohols accounted for 14.1 % of the total volatile compound content. The phytol content (32.4 ± 3.0 μg/kg) was much higher than that of other alcohol compounds, followed by (*Z*)-2-hexenol (11.6 ± 1.8 μg/kg), which is formed from α-linoleic acid (Ho et al., 2015). Phytol originates from the hydrolysis of chlorophyll and is a principal precursor for tocopherol biosynthesis (Lira et al., 2023; Romer et al., 2024). Photosynthesis relies on the absorption of light by chlorophyll. However, excessive light energy leads to lipid peroxidation, which damages the cell membrane, while tocopherol can protect membrane integrity by inhibiting lipid peroxidation (Foyer & Noctor, 2005; Ledford & Niyogi, 2005). Therefore, the high phytol content detected in vine tea might be attributed to the plant's photoprotective effect against high-intensity ultraviolet radiation.

Many terpene compounds were detected in this study, but most of them were in small amounts and only accounted for 12.5 % of the total volatile compounds. Among them, spathulenol showed a significantly high content (14.3 ± 2.6 μg/kg), which is widely found in various medicinal plant essential oils, such as *Schinus molle* (Silva et al., 2023), *Myrciaria tenella* (Apel et al., 2010), and *Salvia mirzayanii* (Javidnia et al., 2002). The high content of spathulenol and its unique herbal-like note may play an important role in the aroma of vine tea (Montalván et al., 2019). In addition, a small amount of esters and heterocyclics with low content were also detected in vine tea, which is consistent with the results reported by Li et al. (2024).

**Fig. 3:**
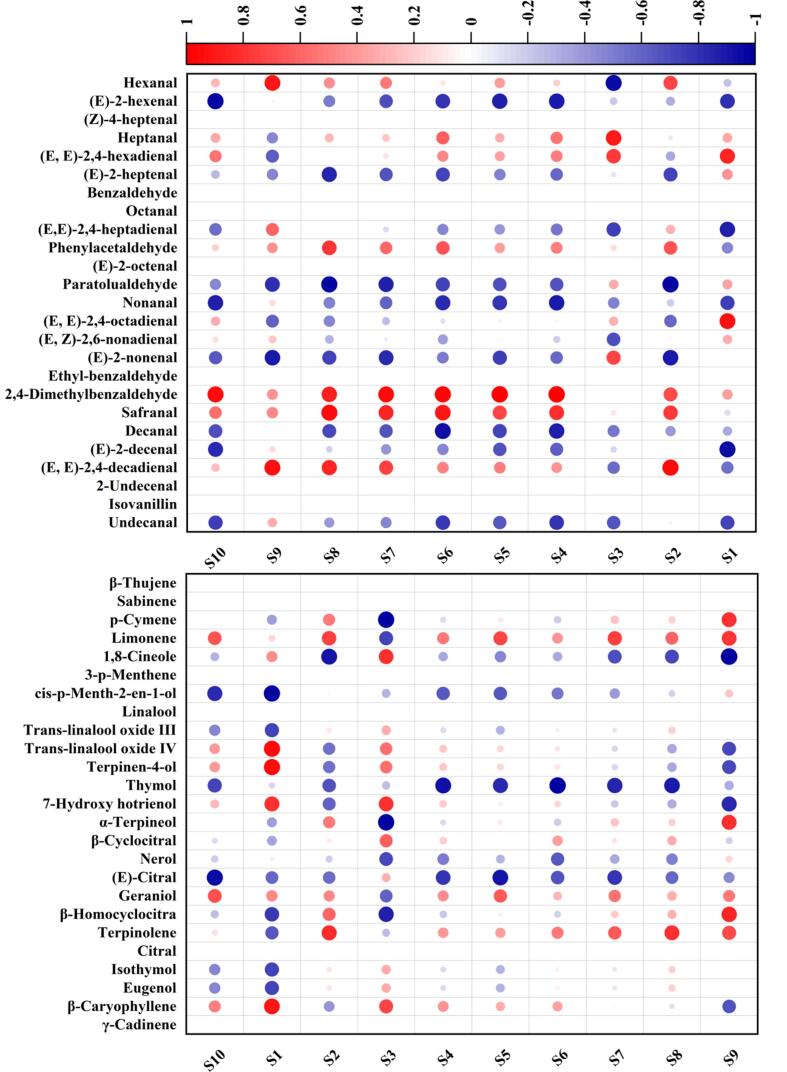

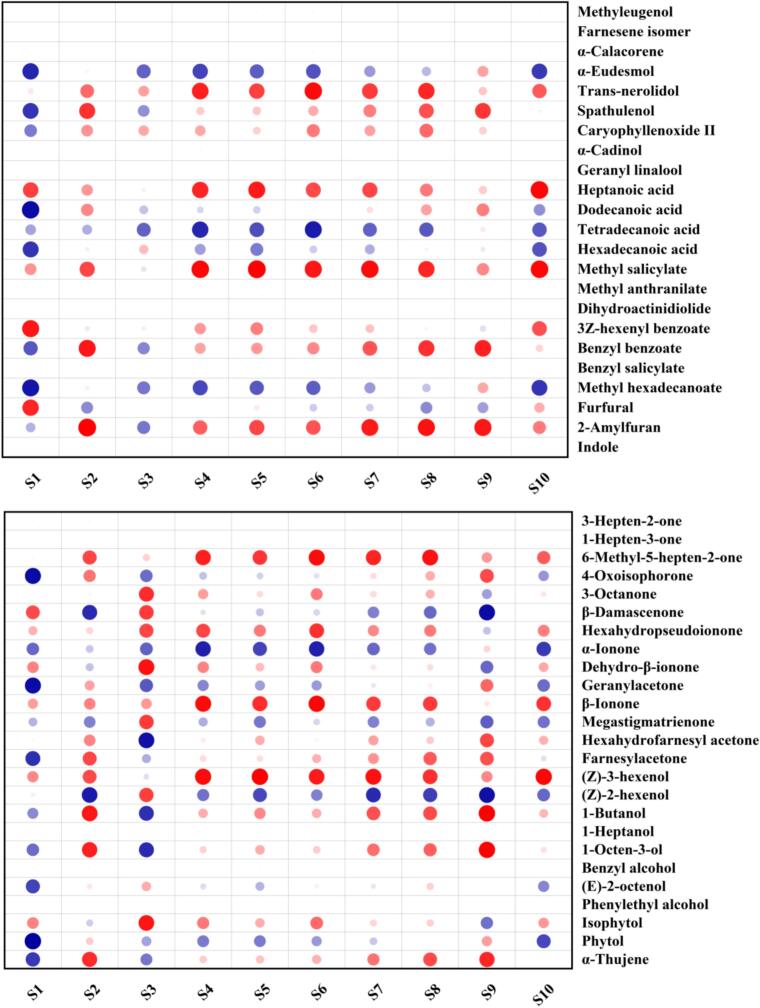
Correlation heatmap analysis between volatile compounds profile and E-nose data of vine tea.

As presented in Fig. 3, correlation heatmap analysis shows that the S1 sensor was highly correlated to (*E*, *E*)-2,4-heptadienal, (*E*, *E*)-2,4-octadienal, *p*-cymene, limonene, α-terpineol and β-homocyclocitra. S2 and S3 sensors correlated highly with (*E*, *E*)-2,4-decadienal and heptanal, respectively. S4-S8 and S10 sensors were highly correlated to 2,4-dimethylbenzaldehyde. Moreover, S6-S8 sensors also exhibited a high correlation to safranal. S9 sensor was highly correlated to hexanal, (*E*, *E*)-2,4-decadienal, trans-linalool oxide IV, terpineol and β-caryophyllene. In addition, some of them also presented moderate or weak correlation to (*E*)-2-heptenal, (*E*, *E*)-2,4-heptadienal, phenylacetaldehyde, paratolualdehyde, nonanal, (*E*, *Z*)-2,6-nonadienal, undecanal, cis-p-menth-2-en-1-ol, trans-linalool oxide III, hotrienol, β-cyclocitral, nerol, geraniol, isothymol, and eugenol. These results suggest that the *E*-nose sensors could effectively distinguish these compounds.

### 3.2 Aroma profile of vine tea

#### 3.2.1 QDA analysis

**Fig. 4:**
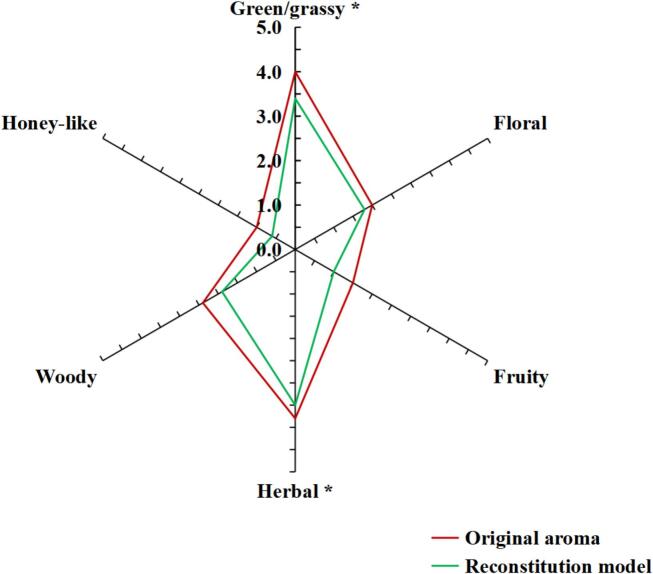
Aroma profiles of original aroma and reconstitution model (*P*<0.05).

QDA analysis was employed to describe and assess the aroma characteristics of vine tea infusion. A total of six different sensory attribute terms were developed and defined in the vine tea aroma, including green/grassy, floral, herbal, fruity, woody and honey-like odor notes (Table 1). Among them, green/grassy and herbal aromas presented strong intensity, followed by a woody aroma, while the honey-like, floral and fruity odor notes were weak (Fig. 4).

**Fig. 5:**
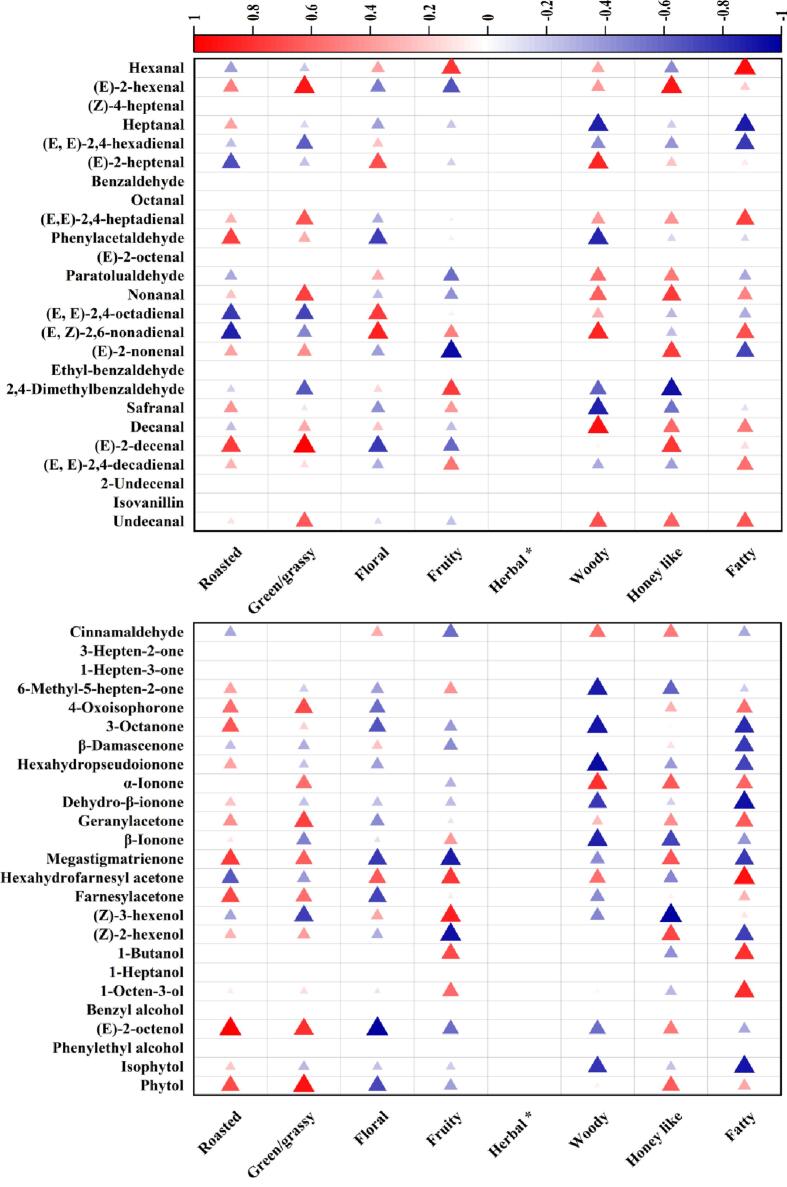

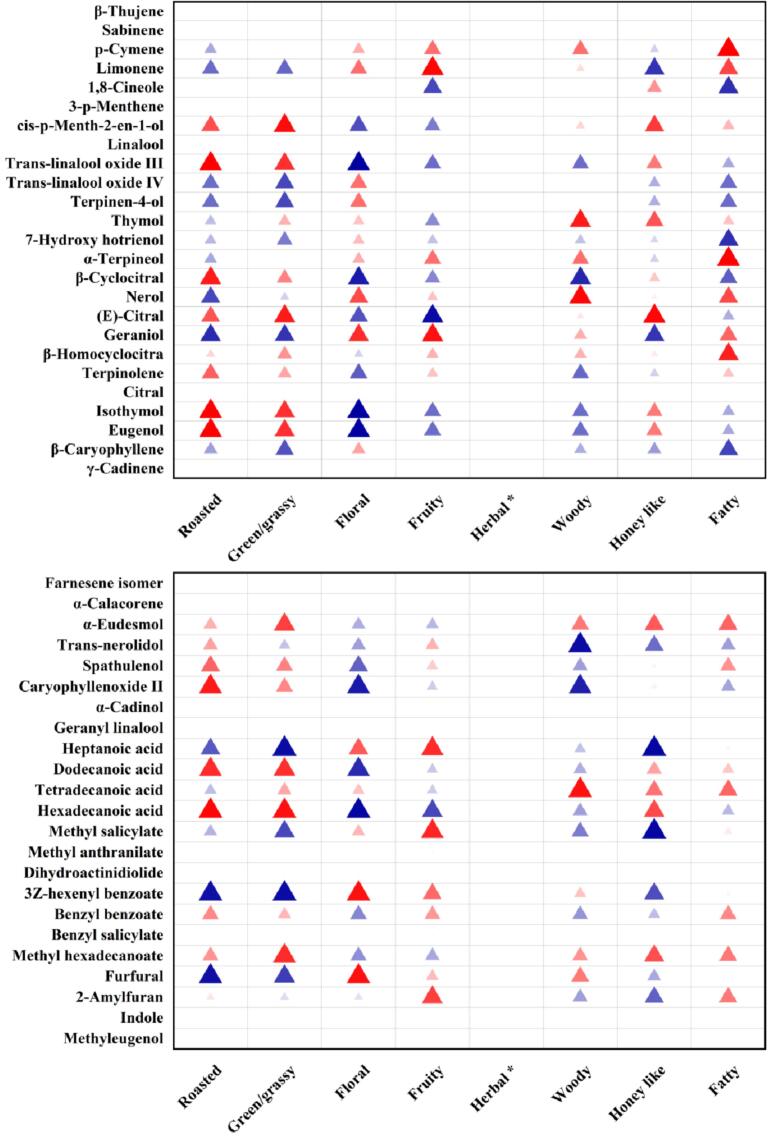
Correlation heatmap analysis between volatile compounds and aroma attributes of vine tea.

**Fig. 6:**
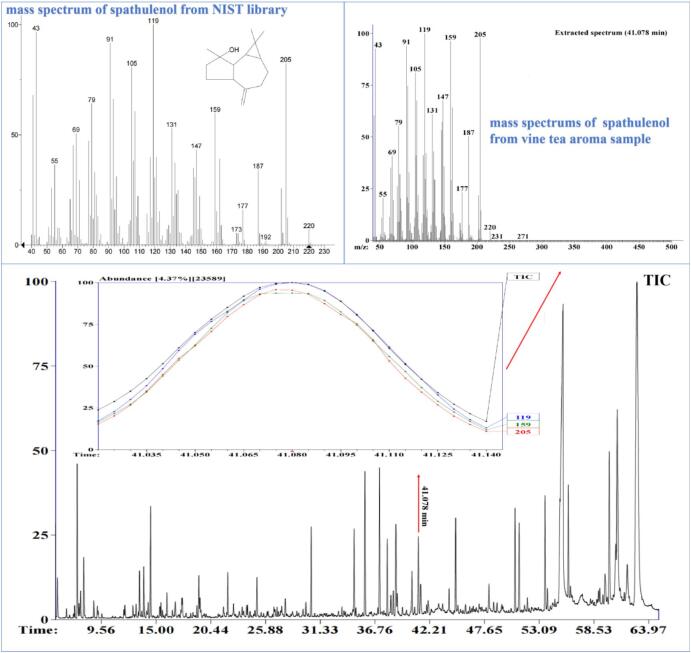
Identification of spathulenol based on GC–MS, RI, and its standard compound.

Correlation analysis between volatile compounds and aroma attributes showed (Fig. 5) that fatty aroma attribute was highly correlated to *p*-cymene, α-terpineol, hexanal and hexahydrofarnesyl acetone, and moderately correlated to β-homocyclocitra, (*E*, *E*)-2,4-heptadienal, 1-butanol, and 1-octen-3-ol. Honey-like aroma attribute showed a high correlation to (*E*)-2-hexanal and (*E*)-citral, and moderate correlation to nonanal, (*E*)-2-nonenal, (*E*)-2-decenal and cis-p-menth-2-en-1-ol. Woody aroma attribute is shown to be highly correlated to (*E*)-2-heptenal, (*E*, *Z*)-2,6-nonadienal, tetradecanoic acid and decanal, and moderate correlation to α-ionone, thymol and nerol. The fruity aroma attribute exhibited a high correlation to limonene, heptanoic acid and methyl salicylate, and a moderate correlation to hexanal, 2,4-dimethylbenzaldehyde, hexahydrofarnesyl acetone, (*Z*)-3-hexenol, geraniol and 2-amylfuran. Floral aroma attribute was moderately correlated to (*E*)-2-heptenal, (*E*, *E*)-2,4-octadienal, (*E*, *Z*)-2,6-nonadienal, geraniol, 3Z-hexenyl benzoate and furfural. Green/grass aroma attribute presented highly correlation to (*E*)-2-hexanal and (*E*)-2-decenal, and moderate correlation to nonanal, (*E*, *E*)-2,4-heptadienal, geranylacetone, (*E*)-2-octenol, phytol, cis-p-menth-2-en-1-ol, trans-linalool oxide III, (*E*)-citral, isothymol, eugenol, α-eudesmol, dodecanoic acid, hexadecanoic acid and methyl hexadecanoate. Roasted aroma attribute was highly correlated to (*E*)-2-octenol, and moderately correlated to phenylacetaldehyde, (*E*)-2-decenal, megastigmatrienone, trans-linalool oxide III, β-cyclocitral, isothymol, eugenol, caryophyllenoxide, dodecanoic acid and hexadecanoic acid. These compounds might play an essential role in the vine tea aroma. For example, (*E*, *E*)-2,4-heptadienal was reported in black, green, and oolong teas and was associated with a fatty odor note (Du et al., 2014). α-ionone, (*E*, *Z*)-2,6-nonadienal and geraniol were related to woody and floral notes, respectively (Lasekan & Lasekan, 2012). (*E*)-2-hexanal, (*E*)-2-decenal and limonene were related to green/grass and fruity notes, respectively (Joshi & Gulati, 2015). Moreover, it could be seen that some compounds played roles in multiple aroma attributes simultaneously, such as hexanal, (*E*)-2-hexanal, nonanal, (*E*)-2-octenol, nerol and isothymol, which suggested that these compounds might lead to the formation of different aroma properties through complex interrelationships (Ferreira, 2012). Although all sensory panelists perceived the herbal aroma attribute in vine tea, the related compounds were not found in the correlation analysis. Therefore, the GC-O and OAVs were performed to understand further the role of these volatile compounds in vine tea aroma. (See Fig. 6.)

#### 3.2.2 GC-O and OAVs analysis

Odorants play an essential role in food aroma. Although many volatile compounds had been detected in vine tea, only a few of them are involved in the formation of aroma (Chen et al., 2019). To identify the odorants responsible for vine tea aroma, the odor attributes and intensity were perceived by GC-O analysis. Also, the retention index and mass spectra were compared with a database built on more than 430 reference aroma compounds we previously identified in different foods or food model systems. In addition, the odorants were further identified using odor activity values, and some unreliable odor-active areas were excluded. Based on these procedures, a total of twelve odorants with intensities ranging from 1.0 (weak) to 5.0 (strong) were detected (Table 3). Among them, (*E*, *Z*)-2,6-nonadienal (cucumber-like and green notes), β-ionone (violet-like and floral notes) and spathulenol (herbal note) were identified with high intensity and OAVs, followed by (*Z*)-4-heptenal (fatty and grassy notes) and β-damascenone (fruity and sweet notes). In contrast, other odorants presented weak intensity. Similar results were also reported by Li et al. (2024). Spathulenol with herbal note was considered an important odorant in the essential oil of *M. myrsinoides* (Montalván et al., 2019), and it may play a key role in the herbal aroma attribute of vine tea.

**Table 3:** Odor perceptions of aroma compounds identified by GC-O analysis.

Odorants	Odor thresholds (ppb)	OAVs	Odor intensity		Odor attributes
(*Z*)-4-heptenal	0.06	35	3.6	Medium	Green
Octanal	0.6	1	1.0	Weak	Fatty
Phenylacetaldehyde	6.3	1	0.8	Weak	Honey
(*E*)-2-octenal	3	1	1.0	Weak	Waxy
(*E*, *Z*)-2,6-nonadienal	0.03	53	4.0	Strong	Cucumber-like
(*E*)-2-nonenal	0.4	2	1.5	Weak	Fatty
Decanal	0.4	2	1.6	Weak	Citrus
(*E*, *E*)-2,4-decadienal	0.16	5	2.2	Weak	Fatty
β-damascenone	0.04	8	3.6	Medium	Fruity
α-Ionone	2.6	6	2.3	Weak	Woody, violet
β-Ionone	0.6	43	4.0	Strong	Woody, violet
Spathulenol	/	/	4.6	Strong	Herbal, earthy

Odor thresholds were taken from Chen et al., 2019, Chen et al., 2020; OAVs, odor activity values, were the concentration ratio to their odor threshold; Odor intensity and attribute were perceived by GC-O; “/,” unknown.

GC-O technique is a valuable tool for identifying odorants, but it ignores the influence of food matrices and the interactions between odorants (Chen et al., 2019). Also, OAVs based on odor threshold only consider the amount of odorants presented in the headspace of the food substrate (Schuh & Schieberle, 2006). Therefore, GC-O and OAVs alone could not wholly explain the relationship between the aroma compounds and vine tea aroma characteristics. For this purpose, the aroma reconstitution and omission experiments were used to further confirm their relationship.

#### 3.2.3 Aroma reconstitution and omission experiments

The aroma reconstitution results are summarized in Fig. 3. The aroma profile of the reconstructed model was similar to the original aroma, which confirmed the critical role of these odorants identified by GC-O and OAVs in vine tea aroma. Moreover, it could be seen that the intensity of sensory attributes in the original aroma was higher than that of the reconstitution model, which might be attributed to the synergistic or additive effects among the low-concentration volatile compounds with similar odor attributes or molecular structures (Niu et al., 2019). For the relationship between the odorants and the aroma attributes of vine tea, most have been reported in other tea beverages except for spathulenol. For example, phenylacetaldehyde was considered an important contributor to the honey-like aroma of black tea (Chen et al., 2019). β-damascenone, β-ionone and α-ionone significantly contributed to green and black tea's fruity and floral flavors (Ho et al., 2015). (*Z*)-4-heptenal, octanal, (*E*)-2-octenal, (*E*)-2-nonenal, decanal and (*E*, *E*)-2,4-decadienal played important roles in the fatty aroma of green, black, and oolong teas, while (*E*, *Z*)-2,6-nonadienal was a representative aroma in various tea beverages, and might be a potential contributor to the green/grass aroma (Chen et al., 2019, Chen et al., 2020; Ho et al., 2015).

**Fig. 7:**
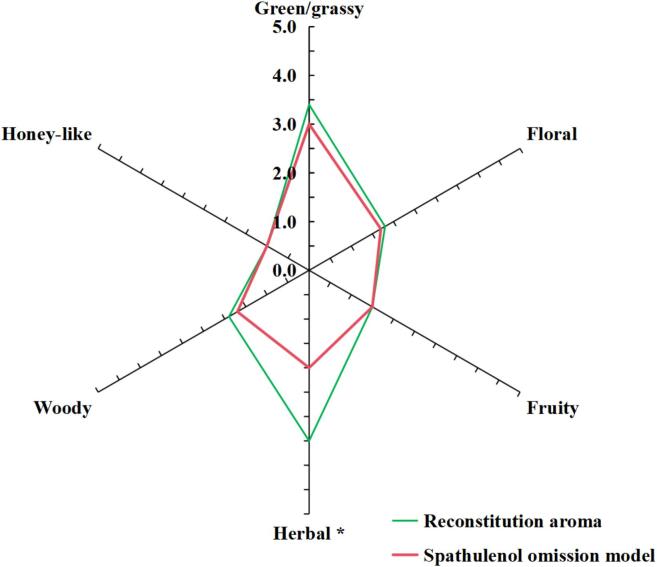
Aroma profiles of the aroma reconstitution model and spathulenol omission model (*P*<0.05).

To further understand the relationship between spathulenol and vine tea aroma, the aroma reconstitution model mentioned above was compared to the omission model, in which only spathulenol was omitted. The results are presented in Fig. 7. Compared to the reconstitution model, the intensity of the herbal odor note was significantly reduced in the spathulenol omission model, indicating that the spathulenol might assist in enhancing the herbal odor in vine tea infusion. In addition, the green/grassy and woody odors were less intense in the omission model than in the reconstruction model, suggesting that spathulenol might also impact the two aromas. To our knowledge, there is no report on the role of spathulenol in vine tea aroma. These results enrich our understanding of the chemical formation mechanism of vine tea aroma.

## 4 Conclusions

A total of 99 volatile compounds were identified in vine tea aroma. Among them, spathulenol and carotenoid-derived volatile compounds were critical characteristics of vine tea's volatile compound profile. The aroma profile of vine tea was characterized by strong green/grassy and herbal aroma, moderated woody aroma, and weak honey-like, floral and fruity odor notes, and twelve odorants were identified as the crucial contributors to the odor notes. Among them, spathulenol played a vital role in the herbal aroma attribute. The potent herbal odor of vine tea and its extracts limits its wide application in the food industry, which may be closely related to the high content of spathulenol in vine tea aroma. Therefore, studies should focus on controlling the formation of compounds with herbal notes by aroma precursors, as well as eliminating the herbal note through process improvements. Our results provided experimental data for the implementation of these strategies.

## CRediT authorship contribution statement

**Xiao Hua Chen:** Writing – original draft, Funding acquisition, Conceptualization. **Yin Ku Liang:** Writing – review & editing. **Fei Yan:** Formal analysis. **Dong Qu:** Resources. **Wei Huang:** Data curation. **Yifan Zhang:** Data curation. **Tian Wan:** Investigation. **Lin Jie Xi:** Funding acquisition. **Ching Yuan Hu:** Methodology, Conceptualization.

## Declaration of competing interest

The authors declare that they have no known competing financial interests or personal relationships that could have appeared to influence the work reported in this paper.

## Data Availability

The data that has been used is confidential.
